# PSMA-Guided Imaging and Therapy of Advanced Adenoid Cystic Carcinomas and Other Salivary Gland Carcinomas

**DOI:** 10.3390/cancers16223843

**Published:** 2024-11-15

**Authors:** Nils F. Trautwein, Andreas Brendlin, Gerald Reischl, Moritz Mattke, Frank Paulsen, Hubert Loewenheim, Lars Zender, Christian la Fougère, Helmut Dittmann

**Affiliations:** 1 Department of Nuclear Medicine and Clinical Molecular Imaging, University of Tübingen, 72074 Tübingen, Germany; 2 Department of Diagnostic and Interventional Radiology, University of Tübingen, 72074 Tübingen, Germany; 3 Department of Preclinical Imaging and Radiopharmacy, Werner Siemens Imaging Center, 72076 Tübingen, Germany; 4 Cluster of Excellence iFIT (EXC 2180) “Image-Guided and Functionally Instructed Tumor Therapies”, University of Tübingen, 72074 Tübingen, Germany; 5 Department of Radiation Oncology, University Hospital, 72076 Tuebingen, Germany; 6 Department of Otolaryngology-Head & Neck Surgery, University of Tübingen Medical Center, 72076 Tübingen, Germany; 7 Department of Medical Oncology and Pneumology (Internal Medicine VIII), University Hospital Tübingen, 72076 Tübingen, Germany; 8 German Cancer Consortium (DKTK), Partner Site Tübingen, 72076 Tübingen, Germany

**Keywords:** salivary gland carcinomas (SGCs), PSMA-RLT, adenoid cystic carcinomas (ACCs), PET

## Abstract

PSMA RLT has primarily been used in the treatment of patients with metastasized castration-resistant prostate cancer (mCRPC). **Background:** The fundamental premise of this theranostic approach is to initially ascertain the presence of the PSMA target through PET imaging, which is generally accepted as a prerequisite for a favorable treatment outcome. PSMA RLT is associated with a low incidence of side effects, making it a favorable option for palliative treatment. In this study, we aimed to evaluate PSMA PET imaging and radioligand therapy for ACCs/SGCs. **Results:** Nine patients with a histologically confirmed disease underwent PSMA PET imaging and were first evaluated using the PROMISE score. The SUVmean and SUVmax of lesions and reference tissue were analyzed in all patients. Patients with a PROMISE score of ≥2 were considered potential candidates for RLT. Three of four patients who were treated with a total of nine cycles (cumulative administered activity 68 GBq [^177^Lu]Lu-PSMA-I&T) were evaluable with a heterogeneous response: two patients showed a progressive disease after three months, while one patient who received four cycles of RLT achieved a stable disease lasting for more than a year. **Conclusions:** PSMA PET and RLT were well tolerated and RLT provides a potential therapeutic option for patients with ACCs/SGCs.

## Simple Summary

Glandular tumors such as adenoid cystic carcinomas (ACCs) are rare malignancies. They often originate from the salivary glands but may also occur in other tissues such as breast, respiratory tract, or Bartholin glands. Primarily, these tumors are often slow growing, but there are only limited systemic treatment options in case of advanced disease, making new therapeutic approaches desirable. One promising option is the theranostic approach with prostate-specific membrane antigen (PSMA) ligands. Positron emission tomography (PET) with radiolabeled PSMA ligands has been developed to image prostate cancer (PC) due to the usually high PSMA expression on the surface of PC cells. A significant milestone was the approval of PSMA-targeted radioligand therapy (RLT) for advanced PC. ACCs and other salivary gland carcinomas (SGCs) often exhibit elevated levels of PSMA expression, generating interest in PSMA RLT as a potential therapeutic option. If successful, this approach could provide a much-needed treatment alternative for these challenging cancers.

## 1. Introduction

SGCs are rare malignancies, accounting for less than 1% of all head and neck cancers. They exhibit a high degree of molecular heterogeneity, with more than 20 distinct subtypes, including ACCs [1]. ACCs most commonly arise in the salivary glands but can also occur in other glandular organ sites such as the breast, paranasal sinuses, and the respiratory tract [2,3]. The five-year survival rate for patients with distant metastasis is approximately 69%, while the ten-year survival rate drops to less than 50% [4,5]. The therapeutic options of ACCs and SGCs include surgery, radiation therapy, and chemotherapy [6]. In recent years, advances in surgery and postoperative radiation therapy have improved overall survival [7,8,9]. However, in the case of recurrent or metastatic disease, treatment options remain very limited, and there is no established first-line systemic therapy. The most commonly used chemotherapies are platinum-based [10]. Prospective data on the chemotherapy response are currently lacking. However, retrospective studies have demonstrated that a combination of taxane- and platin-based chemotherapy could be an option [11]. One study demonstrated that docetaxel plus cisplatin might be superior, showing a prolonged progression-free survival (PFS) compared to paclitaxel plus carboplatin [12]. Nevertheless, the median PFS was only 7.2 months with docetaxel/cisplatin, which highlights the relative resistance of ACC to platin-based chemotherapy. One potential explanation is the upregulation of the ribosomal protein RPS3, which may play a significant role in ACC invasion and chemoresistance [13]. Accordingly, more therapeutic options are under investigation. Recently, several molecular targeting agents have been investigated in phase II studies, with the most promising trials involving tyrosine kinase inhibitors (TKIs) such as Axitinib or Lenvatinib for patients with metastasized ACCs [14,15,16]. However, these studies showed a limited median PFS of 5.7 to 17.5 months. Thus, further therapeutic options for advanced ACCs and SGCs are needed.

In recent years, PSMA PET has revolutionized the management of patients with PC, and PSMA-based RLT has emerged as a promising approach for the treatment of metastasized castration-resistant PC. PSMA-based RLT involves the delivery of therapeutic radionuclides, such as ^177^Lu or ^225^Ac, directly to PSMA-expressing cells [17,18,19,20,21]. Following the pivotal Phase III Vision trial in PC and subsequent approval by both the FDA and EMA, PSMA RLT has become an important part of the therapeutic landscape. PSMA represents a near ideal tumor target due to its relatively low expression in normal human tissue [22]. However, benign and malignant salivary gland tumors and ACCs have shown increased levels of PSMA expression [23,24]. Due to its targeted action, RLT has few side effects and is therefore well-suited for patients in a palliative setting [25].

Moreover, the first studies have already highlighted PSMA PET as a potential diagnostic novelty for ACCs and SGCs [26,27].

The primary objective of our initial study was to determine the PSMA expression levels in SGCs and ACCs. This was achieved by using PSMA PET to explore their eligibility for potential PSMA-targeting RLT. Our subsequent aim was to assess both the occurrence of adverse effects and the therapeutic outcome, by monitoring the safety and response of the first patients undergoing RLT.

## 2. Materials and Methods

### 2.1. Study Cohort

Our PET cohort study database was screened for patients with ACC and SGC, who underwent a diagnostic PSMA PET scan. Written informed consent was obtained from all patients prior to the PET examination. This study was approved by the local ethics committee of our university hospital (decision 497/2023BO2).

### 2.2. [^18^F]PSMA-1007 and [^177^Lu]Lu-PSMA-I&T

[^18^F]PSMA-1007 was synthesized using a GE TRACERlab MX (GE Medical Systems, Liège, Belgium). Reagent kits, the unprotected PSMA-1007 precursor (GMP quality), and the PSMA-1007 reference standard were obtained from ABX (ABX, Radeberg, Germany). Quality control was performed according to the corresponding Ph. Eur. monograph #3116. For [^177^Lu]Lu-PSMA-I&T preparation, PSMA I&T acetate (GMP) was purchased from ABX, and [^177^Lu]LuCl_3_ in n.c.a. quality was provided as EndolucinBeta^®^ (ITM Pharma Solutions GmbH, Garching, Germany). Every batch of [^177^Lu]Lu-PSMA I&T was analyzed for appearance, identity, pH, chemical and radiochemical purity (HPLC, TLC), endotoxins, and sterility. Both tracers were prepared according to German Drug Law §13 2b, preparation under responsibility of the prescribing and treating physician.

### 2.3. PSMA PET Image Acquisition

PET was performed using a Siemens Biograph Vision Quadra (Siemens Healthineers, Knoxville, TN, USA), a Biograph mCT (Siemens Healthineers, Knoxville, TN, USA), or a Biograph mMR^®^ (Siemens Healthineers, Knoxville, TN, USA). Values are presented as the average and the standard deviation. PET imaging was performed for 75 min (±15 min) after injection of 218 MBq (±47 MBq) [^18^F]PSMA-1007.

### 2.4. [^177^Lu]Lu-PSMA-I&T SPECT/CT Acquisition and Reconstruction

Images were acquired 24 h after administration of [^177^Lu]Lu-PSMA-I&T using a dual-head γ-camera (NM/CT 670pro; GE Healthcare, Chicago IL, USA) equipped with medium-energy collimators. Whole-body planar acquisition was performed at a bed speed of 20 cm/min, along with 2 or 3 SPECT/CT bed positions to cover the field of view from the vertex to the mid-thigh. SPECT data were collected over a 180° arc, with 6° steps and 20 s acquisition per step. The reconstruction employed a 3-dimensional ordered-subset expectation maximization algorithm, incorporating CT-based attenuation correction, scatter correction, and resolution recovery. The CT utilized low-dose settings of 30 mA and 120 kV.

### 2.5. Imaging Analysis

PET and posttherapeutic SPECT images were analyzed using a dedicated software tool (Affinity Hybrid Viewer^®^; Version 3.0.5; Hermes Medical Solutions, Sweden).

For PET, the semiquantitative assessment of tracer uptake was performed as follows: For tumor lesions, SUVmax and SUVmean (using a 50% maximum threshold) were measured on baseline PET scans. To reduce over-representation, a maximum of 5 lesions per organ site were analyzed in patients with multifocal or disseminated disease. In order to ensure optimal traceability, the most and least PSMA-expressing lesions were consistently selected, while the remaining up to three were randomly chosen. Reference organ uptake was assessed by measuring SUVmax and SUVmean in a spheric volume of interest with a diameter of 1 cm (thoracic aorta, spleen) and 3 cm (liver).

The PSMA-expression score was assessed visually using the PROMISE criteria [28]. The treatment response was assessed by using the RECIST 1.1 criteria on CT or MRI scans.

On posttherapeutic SPECT/CT scans, the same up to 5 representative lesions per organ site were analyzed, using a 50% maximum threshold, and were normalized to spleen uptake. The spleen was selected as the reference organ, as the liver was deemed non-suitable due to the presence of organ metastases in one patient. Additionally, the spleen is regarded as the optimal reference organ for tracers with liver-dominant excretion such as [^18^F]PSMA-1007, as outlined in the PROMISE criteria [28].

### 2.6. [^177^Lu]Lu-PSMA-I&T RLT Patient Selection and Treatment Administration

Patients with a PSMA score of 2 or 3 were considered for PSMA RLT. The indication for therapy was discussed and decided by a multidisciplinary tumor board. All patients underwent renal scintigraphy and blood sampling to ensure adequate bone marrow and renal function. PSMA RLT was then performed according to the joint guideline of the European Association of Nuclear Medicine and the Society of Nuclear Medicine and Molecular Imaging [29]. This applies except for the treatment interval, where we chose a slightly prolonged 8-week interval between cycles of PSMA RLT due to missing data on safety in patients with ACCs or SGCs. Blood parameters (hemoglobin; leukocytes, neutrophils, lymphocytes, and platelet counts; creatinine; alkaline phosphatase; gamma-glutamyl transferase; lactate dehydrogenase; aspartate aminotransferase; and alanine aminotransferase) were evaluated before and during treatment cycles. Adverse events were graded according to the Common Terminology Criteria for Adverse Events (CTCAE v5.0). The treatment response was assessed using the RECIST 1.1 criteria on CT or MRI scans. The image analysis was performed in the joint consensus of at least 2 experienced readers. Progression-free survival (PFS) was defined as the interval from the start of treatment until death of any cause or progressive disease.

### 2.7. Statistical Analysis

Statistical analysis and illustration were performed using GraphPad Prism version 10.3 for Windows (GraphPad Software, San Diego, CA, USA). The data distribution was tested using a Shapiro–Wilk test. Normally distributed variables were expressed as the mean ± standard deviation, and non-normally distributed variables were expressed as the median and interquartile range (IQR). Data analysis ensued using a mixed-effects model with Greenhouse–Geisser correction in case of violation of sphericity. Post hoc tests were performed for the PROMISE subgroups (<2; ≥2). A two-stage step-up correction following the method of Benjamini, Krieger, and Yekutieli was utilized to counteract a type 1 error increase in the multiple comparisons. An adjusted *p*-value ≤ 0.05 indicated statistical significance.

## 3. Results

### 3.1. Patient Characteristics

**Figure 1:**
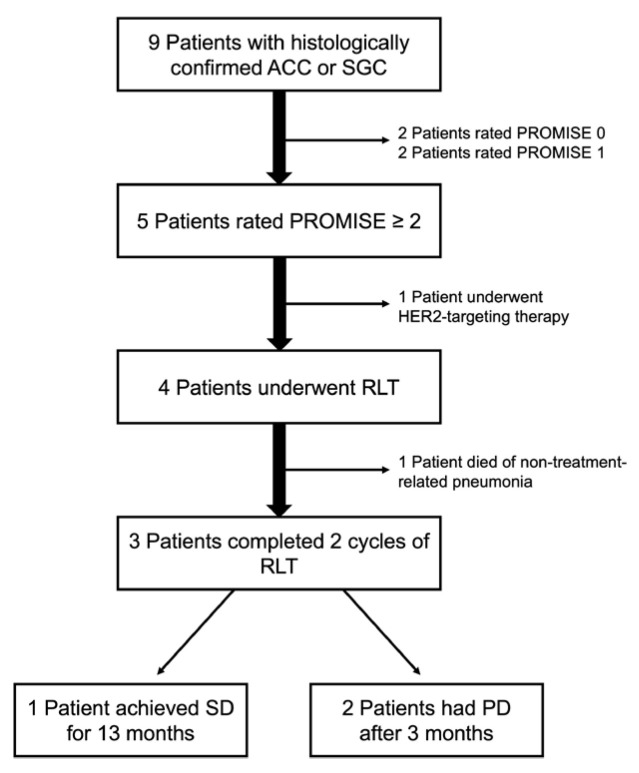
Study flow diagram.

In this study, nine patients (seven females and two males) with ACCs or SGCs who underwent PSMA PET/CT were included. The initial diagnosis of the disease had already been made in these patients years ago. Therefore, all patients were heavily pretreated, and PSMA PET was performed for restaging and to evaluate the option of RLT. A flow diagram that outlines the study design and patient selection is provided in Figure 1.

### 3.2. PSMA PET

**Figure 2:**
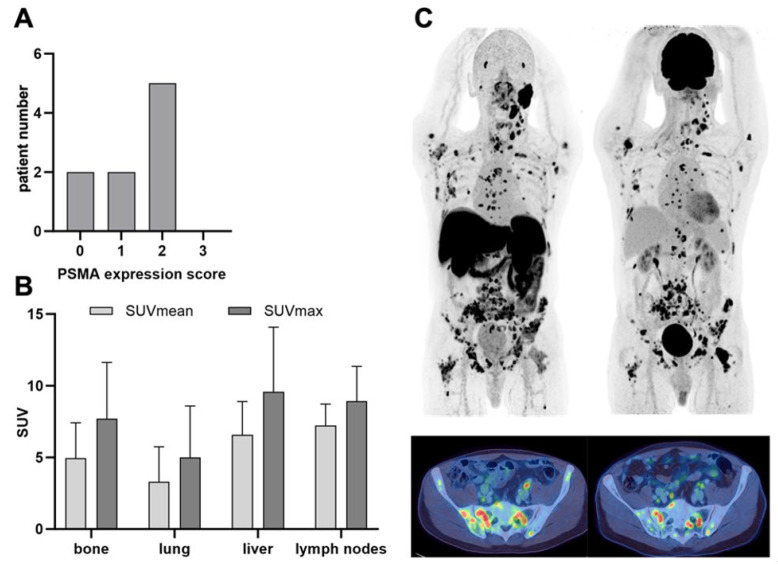
(**A**) PSMA expression score for all 9 patients as determined by PROMISE. (**B**) SUVmean and SUVmax values for all metastases. (**C**) [18F]PSMA-1007 PET (left hand side) and corresponding [18F]FDG PET (right hand side) of a patient with disseminated metastases from an adenocarcinoma of the parotid gland. Maximum-intensity projection (MIP) on the upper row and fused axial PET/CT images of the pelvis are shown.

The PSMA expression score was evaluated for all patients: 0 (n = 2), 1 (n = 2), 2 (n = 5), 3 (n = 0) (Figure 2A). Six patients showed bone metastases with a mean SUVmax of 8.9 (standard deviation (SD) ± 3.1), six patients had lung metastases with a mean SUVmax of 5.7 (SD ± 4.0), two patients had liver metastases with a mean SUVmax of 9.6 (SD ± 4.5), and two patients showed lymph node metastases with a mean SUVmax of 8.9 (SD ± 2.4) (Figure 2B). Figure 2C displays an example of a patient with disseminated disease and intensive uptake of [^18^F]PSMA-1007 (PSMA score 2). This particular patient received an additional [^18^F]FDG PET/CT that revealed an intensive glucose metabolism of the respective tumor lesions.

**Figure 3:**
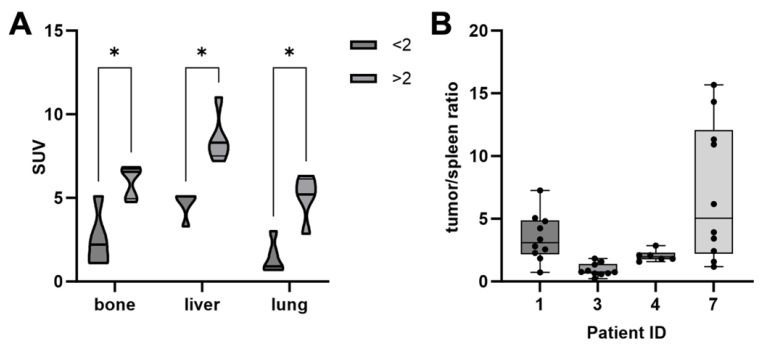
(**A**) Comparison of the SUVmean between tumoral PSMA expression scores of <2 and ≥2 in PET/CT, * *p* < 0.05. (**B**) Tumor/spleen ratio in the posttherapeutic [^177^Lu]Lu-PSMA-I&T SPECT/CT per lesion and patient.

In addition, the SUVmean was analyzed for all patients. Obviously, there was a significantly higher tracer uptake in patients with a PSMA score ≥ 2 (Figure 3A). In these patients, the SUVmean of bone metastases was 6.0 (SD ± 1.8), lung metastases 5.2 (SD ± 1.8), liver metastases 8.5 (SD ± 1.5), and lymph node metastases 7.2 (SD ± 1.5). In patients with a PSMA score < 2, the mean bone SUVmean was 2.9 (SD ± 2.5), lung metastases 1.6 (SD ± 1.4), and liver metastases 4.6 (SD ± 0.8). In the cohort of patients with a PSMA score of <2, there was no evidence of lymph node metastases.

### 3.3. PSMA RLT

Only patients with a PSMA score ≥ 2 were considered for PSMA RLT (Table 1). In total, four patients were treated with nine cycles of RLT (total activity 68 GBq [^177^Lu]Lu-PSMA-I&T). One of these patients died of non-treatment-related pneumonia during the interval between the first and planned second cycle. In this patient, there was no significant decrease in leukocytes.

**Figure 4:**
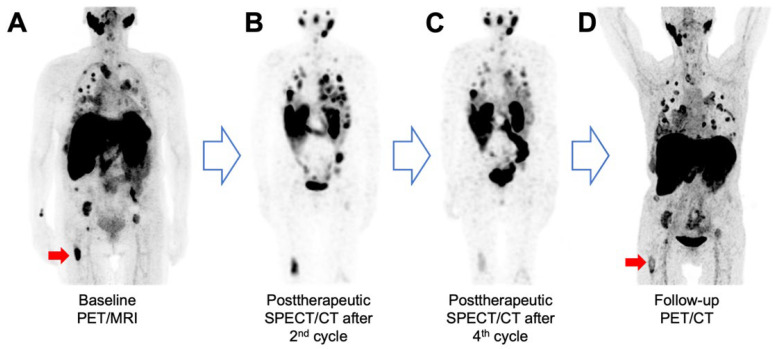
A 60-year-old female patient (ID 1) with an adenocarcinoma of the submandibular gland and a progressive disease under irinotecan combined with an ATR-inhibitor underwent a [^18^F]PSMA-1007 PET/MRI scan. (**A**) The MIP revealed increased PSMA expression in the disseminated lung and bone metastases (PSMA score 2). Posttherapeutic SPECT/CT after two (**B**) and four cycles (**C**) shows the intratherapeutic distribution of [^177^Lu]Lu-PSMA-I&T. (**D**) MIP from the posttherapeutic restaging PET/CT demonstrates a stable disease with decreasing PSMA expression in some metastases (red arrow).

At restaging after receiving two cycles each, two additional patients showed a progressive disease. The fourth patient showed a stable disease, according to RECIST 1.1, on PSMA PET/CT performed for restaging after four cycles. In the latter case, moderate shrinkage and decreased PSMA expression were observed in some of the metastases, for example, in the right femur (Figure 4). In addition, some lung metastases showed a moderate shrinkage in size. In the subsequent follow-up examinations, there was no progressive disease until after 13 months. This patient developed grade 1 anemia and thrombocytopenia following RLT. The other patients had no treatment-emergent side effects. The detailed characteristics of patients undergoing RLT are provided in Table 2.

### 3.4. Post Therapeutic SPECT/CT

Tumor lesions showed an elevated accumulation of [^177^Lu]Lu-PSMA-I&T in the posttherapeutic SPECT/CT images corresponding to the PET/CT findings in all four treated patients (Figure 2B,C). Notably, the uptake intensity as normalized to the reference tissue (spleen) showed a considerable range—both intra- and interindividually (Figure 3B).

## 4. Discussion

Treating metastatic ACCs and SGCs is challenging, due to limited options and the significant side effects associated with most systemic treatments [30]. In addition, disease control is often short-lived [14,15,16]. Therefore, further effective therapeutic options with reduced side effects are needed for these patients.

Recently, PSMA RLT has revolutionized the landscape in metastatic castration-resistant PC by providing an alternative treatment to taxane-based chemotherapy [31,32]. Due to the physiological PSMA expression of salivary glands, the associated carcinomas represent a potential target group for this novel therapy [33].

In this study, we evaluated nine patients with ACCs or SGCs for potential PSMA RLT. PET imaging demonstrated a high PSMA expression score of ≥2 in five patients, and four of them subsequently underwent PSMA RLT. All these patients had received extensive prior treatment. This may explain why none of the patients met the criteria for a PROMISE score of 3. The expression appears to be more heterogeneous than in PC; however, further patient examinations are needed for a comprehensive assessment. In our case series, the treatment was well tolerated by all patients with no severe adverse events (≥grade 3). This adverse event rate is comparable to that reported in studies on PSMA RLT in PC [34,35]. An example is the prospective Vision trial in which 529 patients received up to six cycles of PSMA RLT. The incidence of grade ≥ 3 adverse events was less than 10% for all adverse events except anemia, which was observed in 13% of patients [31]. In these studies, on RLT in PC patients, xerostomia has been demonstrated as a common side effect due to a considerable irradiation dose caused by physiologically high PSMA expression in the salivary glands. This could be a long-term issue also in ACC/SGC patients, especially in those who have previously undergone salivary gland cancer surgery or external beam irradiation.

Disease control with a progression-free interval of more than one year was achieved in one of our patients who received four cycles of RLT. Unfortunately, one patient died prematurely from an infection unrelated to the treatment. Although there was no relevant decrease in leukocytes, this case highlights the importance of monitoring infectious diseases in heavily pretreated patients.

Some preliminary studies have also indicated that PSMA RLT could be a viable option for non-PC patients. Klein Nulent et al. reported the first results of PSMA RLT in salivary gland malignancies, noting a stable disease in two out of six patients [36]. In addition, Civan et al. provided an initial RLT dosimetry in salivary gland malignancies [27]. Taken together, these first results indicate that the treatment response appears to be less favorable than that observed in PC. On the other hand, some patients, such as patient ID1, have achieved at least one year of disease control. This might indicate a possible favorable outcome for individual patients as compared to chemotherapy, which demonstrated a median PFS of 2.8 to 7.2 in SGC [11,12]. RLT may be a viable alternative to tyrosine kinase inhibitors like axitinib, which showed a PFS of 10 months. Further studies are needed to evaluate this hypothesis [16]. Of note, Civan et al. demonstrated that some metastases received only relatively low irradiation doses under PSMA RLT [27]. Ligand washout might be a factor that could limit the achievable tumor dose despite the intense PSMA targeting on PET scans as demonstrated in non-PC carcinoma [37]. Thus, optimized dosimetry with multi-timepoint imaging could improve patient selection. Other options for improving RLT for ACCs/SGCs may include the use of alpha emitters or PSMA ligands with a slower clearance from blood. A study to assess a slower blood clearance agent in ACCs, using [^68^Ga]-PSMA-617 for imaging and potentially [^177^Lu]-EB-PSMA-617 for RLT, is already recruiting patients (NCT04801264). Further improvements in patient selection will be necessary for the broader implementation of RLT in glandular tumors. Prospective studies are needed to further establish this treatment option for ACCs and SGCs. It would be important to test PSMA RLT against platinum-based chemotherapy. Furthermore, an additional FDG-PET could help to improve patient selection as demonstrated in the TheraP study [38].

Limitations: preliminary small cohort study, lack of a control group as there is no standard treatment in advanced ACCs/SGCs.

## 5. Conclusions

PSMA RLT was well tolerated and provides a potential therapeutic option for patients with ACCs/SGCs. In this small cohort, disease control was achieved in one patient for more than one year. In ACCs and SGCs, the PSMA PET results show heterogeneous expression levels. In the current study, only about 50% of patients had a PSMA expression score considered sufficient for RLT. This highlights that pretherapeutic PSMA PET is mandatory to evaluate RLT in ACCs and SGCs and that the PROMISE score can aid patient selection. However, the very heterogeneous results in this small cohort demonstrate the need for further stratification. In addition, further prospectives studies are required.

## Figures and Tables

**Table 1:** Patient characteristics of all PSMA PET patients.

Patient ID	Gender	Age	Years from Initial Diagnosis	Histology	Tissue of Origin	Surgery	Radiotherapy	Systemic Therapies
1	f	60	10.3	Adenocarcinoma	Submandibular gland	Yes	Yes	Doxorubicin + Cisplatin + Cyclophosphamide; Vinorelbine; Paclitaxel; Carboplatin + Gemcitabine; Irinotecan + ATR-Inhibitor
2	f	51	23	ACC	Parotid gland	Yes	Yes	Cabozantinib
3	f	64	3.9	Acinic cell carcinoma	Parotid gland	Yes	Yes	Cisplatin+ 5FU; Trastuzumab + Docetaxel; Doxycycline + Cisplatin + Cyclophosphamide
4	f	34	5.1	ACC	Bronchial gland	No	Yes	Nivolumab + Ipilimumab; Cisplatin+ Paclitaxel; Carboplatin + Paclitaxel; Sorafenib,
5	f	35	3.5	ACC	Mammary gland	Yes	Yes	Carboplatin + Paclitaxel; Cisplatin+ Doxorubicin + Cyclophosphamide; Lenvatinib
6	f	71	16	ACC	Greater vestibular gland	Yes	Yes	Gemcitabine + ATR-Inhibitor
7	m	58	9.8	ACC	Parotid gland	Yes	Yes	Cisplatin + 5FU, Cetuximab; Nivolumab; Docetaxel; Lenvatinib; Cisplatin + Doxorubicin
8	f	61	13.7	ACC	Paranasal sinus	No	Yes	Lenvatinib
9	m	72	3.2	Adenocarcinoma	Parotid gland	Yes	Yes	Cyclophosphamide + Doxorubicin+ Cisplatin; Docetaxel + Trastuzumab

**Table 2:** Detailed PSMA RLT patient information and therapeutic response.

Patient ID	Tumor Sites	Promise Score	Cycles	Total Activity (MBq)	RECIST Response	Time to Progression (mon.)
1	bone, lung	2	4	30.289	SD	13
3	lymph nodes, bone	2	1	7.753	NA	1
4	lung, liver	2	2	14.911	PD	3
7	bone, lung	2	2	15.072	PD	3

## Data Availability

The datasets used and/or analyzed during the current study are available from the corresponding author upon reasonable request.
